# Interventions to improve the detection of depression in primary healthcare: systematic review

**DOI:** 10.1186/s13643-023-02177-6

**Published:** 2023-02-24

**Authors:** Kassahun Habtamu, Rahel Birhane, Mekdes Demissie, Abebaw Fekadu

**Affiliations:** 1 School of Psychology, College of Education and Behavioral Studies, Addis Ababa University, Addis Ababa, Ethiopia; 2 Department of Psychiatry, College of Health Sciences, Addis Ababa University, Addis Ababa, Ethiopia; 3 School of Nursing and Midwifery, College of Health Sciences and Medicine, Haramaya University, Dire Dawa, Haramaya Ethiopia; 4 Centre for Innovative Drug Development and Therapeutic Trials for Africa (CDT-Africa), Addis Ababa University, Addis Ababa, Ethiopia; 5 Global Health & Infection Department, Brighton and Sussex Medical School, Brighton, UK; 6 Department of Psychological Medicine, Center for Affective Disorders, Institute of Psychiatry, Psychology and Neuroscience, King’s College London, London, UK

**Keywords:** Intervention, Depression, Primary healthcare, Detection, Recognition, Systematic review

## Abstract

**Background:**

Several studies have been conducted on the effect of interventions on the detection of depression in primary healthcare (PHC). Systematic reviews have also been done on the effectiveness of separate interventions. However, systematic reviews are not done on the comparative effectiveness of several interventions. This study, therefore, aimed at synthesizing the global evidence on the effectiveness of interventions to improve the detection of depression in PHC.

**Methods:**

We searched PubMed, Embase, PsycINFO, Web of Science, Cochrane Database of Systematic Reviews, Global Index Medicus, African Index Medicus, and African Journals Online, from the inception of the databases to until the 4th week of April 2020. We also searched references of the included articles. We included randomized trials, cluster randomized trials, or quasi-experimental studies, which evaluated the effectiveness of an intervention to improve detection of depression in the PHC setting. Two of the review authors independently extracted data from the included studies. The methodological quality of the included studies was assessed using the Assessment Tool for Quantitative Studies developed by the Effective Public Health Practice Project. The protocol for the review was registered on PROSPERO (CRD42020166291).

**Results:**

Of 23,305 records identified, we included 58 articles in the review. Diverse types of interventions were evaluated to improve clinician diagnosis of depression in the PHC setting. Interventions related to implementation of guidelines, screening with feedback, educational interventions which incorporated active learning and clinical practice, and disclosure of screening results were found to be mostly effective. Interventions which combined education, screening, and feedback were particularly more effective. Most of the included studies were weak or moderate in their methodological quality.

**Conclusions:**

Our review indicates that implementation of a single type of intervention does not improve the detection of depression in PHC. Combining aspects of each type of intervention which are more effective may be useful. Education and training interventions which include more simulation and role playing are found to be effective over time. Most of the studies conducted in the area are from high-income countries and are weak in their methodological quality. There is need to conduct more number of studies in low-income settings.

**Supplementary Information:**

The online version contains supplementary material available at 10.1186/s13643-023-02177-6.

## Background

Depression is projected to be the second leading contributor to the global burden of diseases worldwide by 2030 [1]. This is due to the high prevalence of depression, ranging from 10 to 25% in women and 5 to 12% in men and its impact on daily functioning and mortality [2, 3]. There is also a predominant increasing trend in depression prevalence overtime [4]. Depression is a leading cause of disability, workplace absenteeism, diminished or lost productivity, and increased use of healthcare resource [5]. In addition, depression is associated with increased mortality across all age groups, decreased quality of life, and increased healthcare cost [3]. Depressive disorders accounted for 40.5% of disability-adjusted life years (DALYs) caused by mental and substance use disorders [6]. It is often co-morbid with other several physical as well as mental health conditions with worse outcomes [7].

The prevalence of depression is particularly high in the primary healthcare setting [8, 9]. In a large international study of participants from 14 countries, 24% of attendees of primary care were found to have depression [10]. Studies from Africa also reported almost similar figures [11]. Depression is one of the most common conditions treated in primary care, and nearly 10% of all primary care visits are depression related [12]. Most patients suffering from depression are treated by their primary care physician [13]. Nevertheless, several studies both from high-income and low- and middle-income countries (LMICs) showed recognition of depression in primary care to be suboptimal [14, 15]. In high-income countries, more than 50% of cases with depression may be unrecognized [16]. The detection rate of depression in LMICs by primary care clinicians is extremely low [11]. A study in rural Ethiopia found that over 95% of patients presenting to primary care with potential depression do not receive a clinical diagnosis of depression [17].

Although detection is not a guarantee for treatment, it is a precondition for a patient with depression to be in the path towards appropriate care [18]. Lower level of detection has also a serious impact on the recent efforts to scale up the integration of mental healthcare in the primary care setting [17]. Hence, understanding the factors that impede detection of depression by primary care staff and addressing these factors are of crucial importance. Despite the challenges with recognition of depression in primary care, there is strong evidence that treating depression improves outcomes and is cost-effective [19]. Thus, there is a need to focus more effort and resources on coordinated, multilevel interventions that would improve the recognition of depression in primary care [20].

Several interventions that address the system level needs and the needs of the clinician as well as addressing patient and family/community level barriers to improving the detection of depression in primary care have been evaluated [16, 19, 21, 22]. These include screening [21], clinician education (educational interventions directed at primary care physicians) [23, 24], guidelines [25], case management [15], collaborative care [26], and stepped care [25]. Previous individual original studies, mostly conducted in high-income countries, found that coordinated interventions, such as screening and the chronic care model, are likely to be most effective to detect depression in the PHC setting [27]. Systematic reviews of effectiveness of different interventions to improve the detection of depression in primary care found that the best strategies are those with complex interventions that incorporated clinician education, case management by nurses, and greater collaboration between primary care providers and mental health specialists [25].

Considerable research has focused on the effect of these methods to improve the detection of depression in primary care [27]. Systematic reviews and meta-analysis have also been done to synthesize the evidence on the effectiveness of separate interventions (e.g., screening) to improve the detection of depression [19, 21]. Nevertheless, to the best of our knowledge, there are no reviews which examined the effectiveness of several interventions together. A systematic review focusing on the types and effectiveness of interventions to improve the detection of depression in primary healthcare would allow to identify which interventions have been evaluated and how effective these interventions are. The review would also highlight gaps in the evidence base.

This systematic review, therefore, aimed to address the following questions:

- 1) What interventions have been tested to improve the detection of depression in primary healthcare?
- 2) How effective are these interventions to improve the detection of depression in primary healthcare?

## Methods

We followed the Preferred Reporting Items for Systematic Reviews and Meta-Analysis (PRISMA) guidelines throughout our review [28]. The protocol for the review was registered on PROSPERO (CRD42020166291).

### Population and scope of review

Studies with adult or adolescent participants (age 15 or over) attending primary care facilities with depression, including major depressive disorder, bipolar depression, masked depression, secondary depression, minor depression, and subthreshold depression, were considered for inclusion in the review. We included all the different types of depression diagnoses, ranging from major depressive disorder to subthreshold depression; however, this has to be ascertained either through clinician diagnosis or using a structured symptom scale with a validated cut of point. A study was included if it examined the effectiveness of an intervention with the aim to improve detection of depression in the primary care setting. The inclusion criteria can be presented with the PICOS (population, intervention, comparator, outcome, and setting) framework as follows:

• P = Adults or adolescents (age 15 or over) attending primary care facilities and have depression
• I = Any intervention with the aim to improve detection of depression in the primary care setting
• C = Any comparator, a study was included if there was no comparator at all.
• O = Change in rate of detection of depression by PHC workers
• S = Study conducted anywhere in the world but in PHC setting

We excluded a study if it has reported only the prevalence of depression or detection rate of depression. We also excluded a study if it was not conducted in a primary care setting (e.g., conducted in a specialist hospital or community setting) even if it has evaluated the effectiveness of an intervention to improve the detection rate of depression. We also excluded a study if it is a review of any kind.

### Outcome of interest

The outcome of interest in this systematic review was improvement of detection of depression in the primary care setting. In this review, detection is understood as the proportion of the number of patients correctly diagnosed as having depression by primary care clinicians compared to a diagnosis using a locally validated screening tool or a confirmatory clinical diagnosis by a mental health expert. Improvement in detection of depression is understood as change in the number of patients diagnosed as having depression by primary care clinicians following the intervention. This can be compared to diagnosis using screening tools, case vignettes, review of medical records, and clinical diagnosis by a mental health expert.

### Types of study to be included

We included randomized controlled trials (RCT), cluster randomized trials, or quasi-experimental studies that involved the following:

• Development of an intervention which aimed to improve the detection of depression at a primary care setting
• Sociocultural adaptation of an intervention that would help to improve the detection of depression in primary healthcare
• Test the efficacy of an intervention to improve detection of depression in primary care
• Evaluate the effectiveness of an intervention to improve detection of depression in primary care

### Search strategies

We searched major databases (PubMed, Embase, PsycINFO, Web of Science, and Cochrane Database of Systematic Reviews) from the inception of the databases to until the 4th week of April 2020. We also searched other databases, such as the Global Index Medicus which include Latin American and Caribbean Health Sciences Literature (LILACS), African Index Medicus (AIM), and African Journals Online (AJOL). References of included studies and authors who have conducted studies on detection and management of depression in primary care were also consulted.

We identified a number of terms to represent the four big terms. To identify the big term depression, we used such terms as depression, depressive disorder, major depressive disorder, minor depression, and bipolar depression. The search terms that were used for detection include detection, detection rate, prevalence, screening, case finding, and diagnosis. For primary health care, we used such terms as primary health care, primary care, health center, and primary hospital. To represent the term intervention, we used the terms intervention, strategies, methods, mechanisms, etc.

Depending on the database, we tried to find standardized terms representing each of our big terms and include them in our search. Then, the terms for depression, detection, intervention, and primary healthcare were combined with the Boolean term “AND.” On the basis of these search terms, detailed search strategy was developed before searching from each database. We reported the complete search strategy for the PubMed database (see Additional file 3).

### Screening and data extraction

Three of the authors of this review (KH, RB, and MD) independently screened the titles and abstracts of 10% of the studies identified from the databases searched and those identified from additional sources. Additional sources were references of included studies identified from the databases and from consultation with authors who conducted research on detection and management of depression in primary care. Then, the rate of agreement to include or exclude these articles among the three screeners based on title and abstract screening was computed. Equal proportion of the rest of the titles and abstracts of the identified studies was screened only by one of the three authors stated above. As the rate of agreement was quite high (more than 90%), we decided the rest of the titles/abstracts to be screened by just one person. When a screener was not sure to include/exclude, they consulted the other two screeners and decided based on consensus. This was done to identify studies that potentially meet the inclusion criteria stated above. Then, the full text of the eligible studies were retrieved and assessed independently for eligibility by two members of the review team. Any disagreement between the two independent screeners over the eligibility of full-text studies was resolved through discussion with and by the recommendation of another member of the review team, who is more senior and experienced. We documented excluded articles and reasons for exclusion.

We developed and used a data extraction form for study characteristics and outcome data. This data extraction form was pilot tested before actual implementation. Two of the review authors independently extract the following data from included studies:

• Author/s
• Study country
• Study design
• Study setting
• Population
• Sample size
• Sample characteristics (gender and age)
• Intervention/s
• Control condition/s
• Length of follow-up
• Outcome/s measured (detection or prevalence)
• Outcome measures
• Summary of results

### Quality assessment

All included studies were assessed for risk of bias independently by 2 researchers. We assessed the methodological quality of the included studies with reference to several criteria, including the methods of selection, allocation, blinding of outcome assessors, dropout, and intervention integrity. Differences of opinion were resolved by a third senior reviewer. The “Quality Assessment Tool for Quantitative Studies” developed by the Effective Public Health Practice Project (EPHPP) [29] was used to assess specific dimensions of the quality of the studies. The tool has eight items which assess selection bias, allocation bias, control of confounders, blinding of outcome assessors, data collection methods, withdrawals and dropouts, intervention integrity, and analysis. The tool allows global rating of “weak,” “moderate,” or “strong” for each included article. An article can be rated as “strong” if poor rating was not made in any of the eight items, “moderate” if poor rating was made in just one of the items, and “weak” if poor rating was made in two or more of the items. For each item, there are specific criteria to rate “good,” “fair,” or “poor.”

We reported the data for six of the eight items. We did not report the data for two of the items (intervention integrity and data analysis). This is because overall rating can be made with the first six items, and we found no difference in the rating of the included articles in terms of these last two items as almost all papers reported good intervention integrity and used the right data analysis. We used the Quality Assessment Tool for Quantitative Studies because it is designed to assess the methodological quality of studies with different quantitative designs: RCT, cluster RCT, and different types of quasi-experimental designs (such as before-after with control group and before-after with no control group).

### Strategy for data synthesis

Narrative synthesis of the findings from the included studies was provided. The synthesis was structured in line with the review questions into the following: interventions developed, adapted, or tested to improve detection of depression, effectiveness of interventions to improve detection of depression, and quality of the studies included in the review.

We summarized key findings in the form of figures, tables, and text. Our original plan was to conduct meta-analysis and network meta-analysis, generate summary effect sizes of interventions, and determine direct as well as indirect effects and rank order interventions in terms of their effect to improve the detection of depression. However, we were not able to do all these because of the heterogeneity of the included studies in terms of several factors, including type of intervention, study design, content and duration of interventions, duration of follow-up, format of intervention delivery, outcome measures, and methods of statistical analysis used to determine effect size.

## Results

**Fig. 1:**
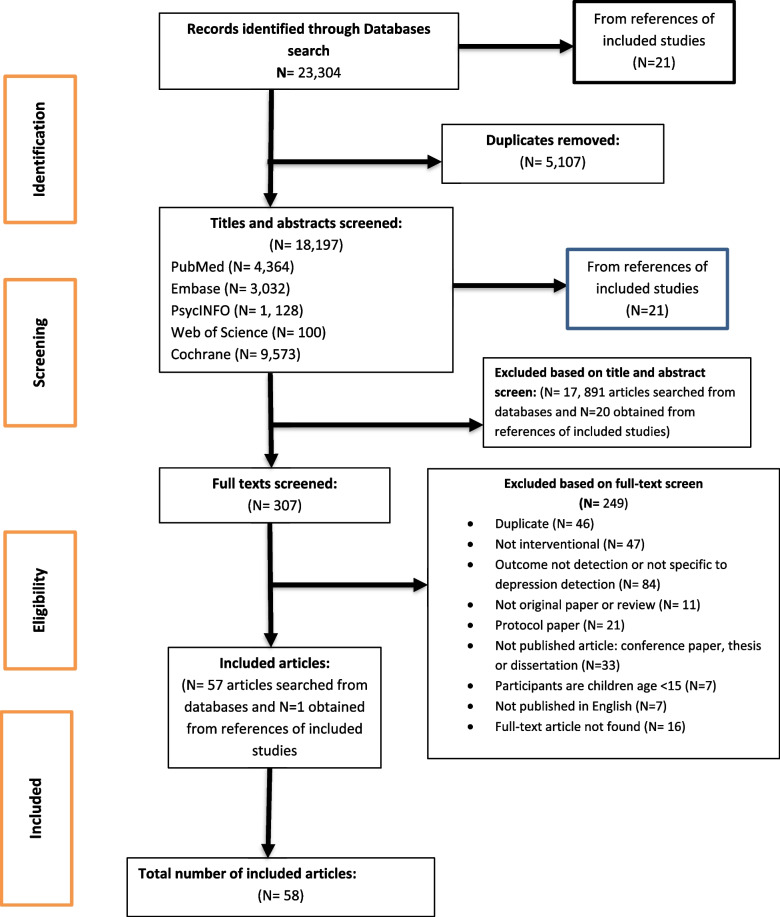
PRISMA (Preferred Reporting Items for Systematic Reviews and Meta-Analysis) flow diagram

We identified a total of 23,304 records from our search of databases. Of these, 5107 were excluded as they were duplicates. Of 18,197 articles, 17,891 were excluded after title/abstract screen as they were not related to depression or detection of depression or they do not involve any kind of intervention, or the studies were not conducted in PHC setting. Of the 306 studies included in full-text screen, 249 were excluded because of several reasons: duplicates, outcome not depression detection, the study does not involve any intervention, not original study, protocol paper, not published article, published not in English language, and full-text article not found. We included one more full-text article from screening 21 articles obtained from the references of the included studies. Hence, 58 articles were included in the final analysis. The PRISMA flow chart which describes the identification, screening, and inclusion process is presented in Fig. 1.

### Characteristics of the included studies

Most of the studies were from high-income countries (*n* = 47/58), particularly from the UK and USA. Only six studies were from low-income countries. In terms of study design, nearly half of the studies (*n* = 28) were before-after with no control group. Twenty-five studies were randomized (18 RCT and 7 cluster RCT). Many of the studies had small sample size; around half of the studies had sample size less than 150. Most of the studies (*n* = 34) used either purposive sampling or convenience sampling to select participants. Five of the studies did not describe the sampling technique they used to select participants.

The included studies in the review were diverse in terms of measuring depression detection (Table 1). While most of the studies used screening tool (*n* = 36), the other studies used case vignettes (*n* = 5), psychiatric interview (*n* = 6), and record review or chart audit (*n* = 11). Most of the studies were weak (*n* = 14) or moderate (*n* = 30) in their methodological quality. Only 14 out of 58 studies were assessed as strong in their methodological quality. For details of characteristics of the included studies, see Additional file 1.

**Table 1:** Summary of the data extracted from the included studies (*n* = 58)

Areas of summary	Categories	Number of studies	Percent
Income level of countries of the included studies			
	High income	47	81.0
	Middle income	5	8.6
	Low income	6	10.3
Study design			
	RCT	18	31.0
	Cluster RCT	7	12.1
	Before-after (pre-post) with control group	5	8.6
	Before-after (pre-post) with no control group	28	48.3
Number of intervention groups			
	One	28	48.3
	Two	24	41.4
	Three	3	5.2
	More than three	3	5.2
Sample size			
	< 100	23	39.7
	100–150	6	10.3
	151–250	6	10.3
	> 250	23	39.7
Types of participants			
	PHC workers	26	44.8
	Patients attending PHC	16	27.6
	PHC workers and patients attending PHC	16	27.6
Sampling technique			
	Purposive sampling	20	34.5
	Convenience sampling	14	24.1
	Random sampling	19	32.8
	Sampling technique not clear	5	8.6
Type of intervention/treatment			
	Clinician training (education)	22	37.9
	Screening	9	15.5
	Feedback (disclosure of screening results)	7	12.1
	Development and dissemination of guidelines/collaborative care package/quality improvement	10	17.2
	Request for anti-depressants	1	1.7
	Combination of interventions (education, screening, and feedback)	9	15.5
Outcome measure			
	Case vignette	5	8.6
	Screening tool	36	62.1
	Psychiatric interview	6	10.3
	Document review (chart audit)	11	19.0
Global quality assessment of studies			
	Strong	14	24.1
	Moderate	30	51.7
	Weak	14	24.1

*RCT* Randomized controlled trial, *PHC* Primary healthcare

### Interventions to improve detection of depression in primary healthcare

Diverse types of interventions were tested to improve the detection rate of depression in the primary healthcare setting. We grouped these interventions into six types. They included the following: (i) clinician training (education), (ii) screening alone, (iii) screening with feedback (disclosure of screening results to clinicians), (iv) combination of education, screening, and feedback interventions, (v) implementation of guidelines/collaborative care packages or quality improvement programs, and (vi) request for antidepressants. A relatively larger number of the studies tested clinician training (education) intervention (*n* = 22/58), followed by implementation of guidelines, collaborative care packages, or quality improvement programs (*n* = 10/58). Nine studies tested the effectiveness of screening intervention. Seven studies evaluated the effectiveness of screening with feedback, whereas another nine studies tried to check for the effectiveness of combining training, screening, and feedback interventions. Only one study evaluated the effectiveness of patient request for antidepressants to improve the ability of primary care clinicians to detect depression. Although different studies evaluated the same type of intervention (e.g., training or screening or feedback), the duration, intensity, content, and format of the intervention differed. The interventions tested in each of the included studies are summarized in Tables 2, 3, 4, and 5.

**Table 2:** Description of interventions and results of studies with clinician training (education) intervention

Citation (author, year)	Description of the intervention	Duration of intervention, length of follow-up	Outcome measures	Estimate of effect/summary result
Adebowale et al. (2014) [30]	Training course (locally developed from the WHO mhGAP intervention guide)	Three days, 2 months (8 weeks)	Case vignettes to assess ability to make accurate diagnosis and to list appropriate treatment recommendations	A total of 92.5% pre-training and 93.8% post training, *P* = 0.200
Alexander et al. (2013) [31]	Training which focused on providing basic information about depression and demonstration of a depression-screening strategy	One hour, 2 months	Self-reported questionnaire and case vignettes	A total of 22.2% in the intervention and 16.7% in the control group (*P* > 0.05)
Andersen et al. (1990) [32]	A single evening seminar focused on the diagnosis, treatment, and referral of selected DSM-III/DSM-IIIR affective and anxiety disorders in primary care medicine)	Three and half hour, 1–8 weeks	Diagnostic knowledge inventory, which includes 18 paragraph-length case vignettes	Mean diagnostic accuracy 1.35 in the intervention group vs 0.97 in the control (*P* < 0.01). Proportion of physicians diagnosing cases correctly in the intervention and control groups at pretest 44.5 vs 53.4 and posttest (54.2 vs 40.9), *P* < 0.12
Bodlund et al. (1999) [33]	Regular consultations on a fortnightly basis (GPs had the opportunity to present cases with manifest or possible psychiatric disorders to the psychiatrist during 90-min sessions). This procedure was complemented with 2–3 training sessions	One year, 1 year	Hospital Anxiety and Depression scale and medical records	Prevalence increased from 3.7 to 4.7% (*P* > 0.05). Cases with clinically diagnosed depression increased from 4.0 to 7.9% (*P* < 0.05). Agreement between HAD diagnosis and clinical evaluation of depressive disorders improved from 20% (*k* = 0.18) to 45% (*k* = 0.54). After 1 year, GPs identified twice as many of the patients that suffered from anxiety or depression
Davidson et al. (2006) [34]	GPs attended a single education session delivered by a psycho-geriatrician. The session covered the diagnosis and treatment of late-life depression and training in the use of the Cornell Scale. Nursing staff attended a session, delivered by a clinical psychologist with expertise in late-life depression	Single education session	Patient file review form, Cornell Scale for Depression in Dementia and Algorithm for the assessment of depression in the elderly	At review, 24% of patients had Cornell Scale scores > 10. A further 32% had Cornell scores between 6 and 9
Fallucco et al. (2019) [35]	A seminar plus a practical clinical skill in assessment and treatment of adolescent depression. Training was led by a psychiatrist and was followed by a 30-min debriefing and question-and-answer period. PCPs received an educational packet including copies of the screening tool, treatment algorithms, and antidepressant medication dosing guidelines	Sixty-minute seminar and 60-min practical clinical skill, 12 months	The EMR was queried for specific procedural codes for depression screening and for any adolescent well-visit with a new diagnosis of major depressive disorder	In the year before the training intervention, 28 of 3150 (0.89%) adolescents were diagnosed with depression compared with 88 of 3958 (2.22%) adolescents seen after training. The odds of receiving a new diagnosis of depression were almost three times higher after training (*OR* = 2.7; 95% *CI* = 1.8–4.2, *p* < .0001)
Garg et al. (2019) [36]	On-site training section of a proposed 1-year-long NIMHANS Extension for Community Healthcare Outcomes training consisted of 2 h of didactic session on a topic, followed by a 2-h visit to the outpatient department	Two weeks, the PCDs were administered the vignettes before the commencement and after the completion of the onsite training program (2 weeks)	Ten case vignettes: each vignette was worth 10 marks, five each for diagnosis and management (a total score of 100)	The post-training score (83.42 ± 10.38) was significantly higher than the baseline score (42.4 ± 23.10), *P* < 0.001. The improvement was significant across all the vignettes
Gomez-Restrepo (2007) [37]	Training was carried out that consisted of a theoretical part and a practical part, focusing on case discussion, role-playing games, and use of vignettes for clinical cases	A day training, 2 months	SSI-CIDI	Before training: *n* = 97/1647 = 5.9% (95% *CI* = 4.8–7.1) and after training: *n* = 196/1832 = 10.64% (*CI* = 9.2–12.06%)
Hannaford (1996) [38]	Educational intervention, details not reported	Three months, 5 months	Hospital Anxiety and Depression (HAD) scale	Before intervention missed diagnosis = 24.1% (124/515) and after intervention missed diagnosis =17.1% (81/475); absolute decrease 7% (95% *CI* = −2.0 to −12.0%), *P* < 0.005
Haddad et al. (2018) [39]	Training was delivered to groups of school nurses according to their service teams, which at the time of the study (2008–2011) were primary care trusts. The training sessions were delivered with an identical program and resources by the same trainers (clinicians and service user), with group sizes of around 10 staff attending	A day training with 4–6 weeks follow-up, 9 months	Vignette method	Sensitivity of depression recognition varied between the trial clusters from 52 to 86.7%; specificity varied between clusters from 39.3 to 57.1%. Specificity differed between groups following the intervention at 3 months (49.3% vs 57.1%, *P* = 0.039) and at 9 months (45.3% vs 52.9%, *P* = 0.001)
Kauye et al. (2014) [40]	PHC workers in the intervention group underwent a training program in mental health using a toolkit originally designed for Kenya	Five days training	The SCID for depression	Before intervention, 0% in intervention and control, and after intervention, 9% in the intervention and 1% in the control
Kick et al. (1999) [41]	The intervention group received instruction in and copies of the AHCPR Quick Reference Guide for depression in primary care. The residents were given copies of the PRIME-MD and taken through a training session utilizing its mood disorder module	Three times, once in a month, 6 months	Center for Epidemiologic Studies Depression Scale	At baseline, the intervention residents knew more DSM-IV criteria for depression (*P* = 0.03) than the control group. At 6 months, the intervention residents still knew more DSM-IV criteria for depression (*P* = .03)
Lin et al. (2001) [42]	Participants in the intervention group were given standard training. It had role plays, and those unable to attend small group sessions study, psychiatrists individually met them	Two hours training, 3 months	ICD classification, no scale mentioned	Before intervention, 1.84 per 100 visits in the intervention and 1.63 in the control; after Intervention, 1.91 in the intervention and 1.68 in the control
Kutcher et al. (2017) [43]	Teachers were trained in the use of a mental health literacy curriculum resource (“The African Guide”) and applied it in their classrooms. Teachers received training in how to identify youth who may be showing signs and symptoms of depression and how to refer them to their local community health clinics. Community health clinic staff received training in the youth depression identification, diagnosis, and treatment	Six months	Kutcher Adolescent Depression Scale and chart review	One-hundred twenty-one youth screened of which 107 (88.4%) screened as potentially positive for depression. Then, in health facilities, 85 youth (71.4%) were diagnosed with depression using the tool
Pond et al. (1994) [44]	A brief structured educational visit to the doctor by an academic detailer, a fellow GP. The academic detailer presents clear, concise, and relevant facts about depression on a one-to-one basis. The detailer attempted to be as interactive as the situation permitted	Six months	GPs diagnosis was compared with diagnosis using screening instruments (GDS, CEI)	GP identification vs GDS-10: kappa increased from 0.06 to 0.16 (*P* = 0.21), and sensitivity improved from 33 to 42%, while specificity remained the same. GP identification vs GDS-13: kappa increased from 0.11 to 0.37 (*P* = 0.03), and sensitivity improved from 36 to 56%, while specificity remained about the same. GP identification vs DSM III-R and ICD-10 diagnoses: kappa increased significantly (*P* = 0.03 and *P* = 0.02, respectively)
Thompson et al. (2000) [24]	Practical guideline to the detection, assessment, and management of depression was provided. Education was provided in two parts. Seminars, in groups of up to 20, were held at the beginning of the intervention year. Each practice received seminars. Teaching was supplemented by videotapes to demonstrate interview and counseling skills, small-group discussion of cases, and role play if appropriate	Four hours seminar plus follow-up education for 9 months depending on the need, 6 weeks and 6 months	Diagnosis by physician was done using a 4-point global scale and HADS	The sensitivity of physicians to depressive symptoms was 37% in the intervention group and 35% in the control group after seminars. A total of 39% in the intervention group and 36% in the control group after education and 34% in the intervention group and 36% in the control group at the end of the study
Van Daele et al. (2015) [45]	Minimal intervention consisting of information, skill training, and discussion. The training was given by a researcher and a staff member of the home nursing organization for small groups of about ten home nurses at a time. It started with a discussion on familiar topics	One-hour session, 3 and 7 months	Using the screening questions formulated by Whooley et al. (1997) and Arroll et al. (2005) and the reporting sheets	In the 3 months following the intervention, home nurses detected 42 depressed patients and family caregivers (*N* = 16 out of 63) in the intervention group) and (*N* = 2 out of 29) in the control group, *P* = 0 038
Vanos et al. (1999) [46]	The training consisted of eight sessions. The A group training included sessions about somatization, sleeping problems, and chronic complaining, plus an introductory and a booster session. In the B group, the introductory and booster sessions were replaced by two sessions on anxiety disorders. Each session followed a similar structure: (1) discussion of the normal practices and difficulties of the trainees; (2) a short lecture by the psychiatrist trainer; (3) illustration with video-taped consultations; (4) introduction of guidelines and protocols for screening, diagnosis, or interventions; (5) practice using various forms of hands-on learning (e.g., role playing); and (6) evaluation	8 sessions 2.5 h each, 1 year	The 12-item version of the GHQ to screen for psychological distress; CIDI-PHC was used for the second-stage baseline assessment of patients and yields ICD-10 diagnosis	Diagnosis of depression (pre 40%, post 48%, *P* = 0.12). There was significant improvement in the A group (pre 35%, post 51%, *P* = 0.03), but none in the B group (pre 45%, post 45%, *P* = 0.99). In adjusted model, there was no overall significant pre-post differences in diagnosis of depression (*OR*: 1.39, *P* = 0.15)
Worrall et al. (1999) [47]	Physicians in the intervention group attended a small educational workshop where they were introduced to the clinical practice guidelines formulated by the Canadian Medical Association for the detection and treatment of depression. Workshops were led by a psychiatrist and an academic family physician	Three-hour educational session and consultation by psychiatrist at a specific time each week, 6 months	Data were collected from physicians record; each patient was rated on a 4-point ordinal scale (4 = severe depression to 1 = absence of depressive symptoms). CES-D was used for assessing patient outcome	Physicians in the intervention group diagnosed 91 new cases of depression (mean 4.1 per physician) and those in the control group diagnosed 56 (mean 2.8 per physician). Fifty-three (93.4%) in the intervention group and 84 (94.6%) in the control group made correct diagnosis
Shirazi et al. (2013) [48]	Educational intervention tailored according to the participants’ readiness to change. Interactive workshop for a small group of GPs at a higher stage of readiness-to-change (“intention”) and interactive large group meeting for those with lower propensity to change (“attitude”). Interactive and multifaceted learning activities were used, such as case illustrations, standardized patients, role playing, buzz groups, programmed lectures, and snowball techniques. Printed materials were given to the participants	Two-day training (12 h education plus 8 h teacher contacts) for both groups and 4 h collaborative small group learning for the intervention group, 2 months after the intervention	Checklist compiled using *Diagnostic and Statistical Manual of Mental Disorders IV* criteria for diagnosis	Mean (SD) of performance regarding diagnosis at intervention group A vs. control group • Pre-intervention 48 (22) vs. 48 (25) and post-intervention 63 (20) vs. 49 (24), *P* = 0.007 Mean (SD) of performance regarding diagnosis at intervention group B vs. control group • Pre 33 (28) vs. 26 (31) and post 49 (35) vs. 22 (25), *P* < 0.001
Vicente et al. (2007) [49]	A brief educational training program for depression designed by the World Psychiatric Association. The World Psychiatric Association training module was administered in a seminar organized by the site coordinator, an experienced psychiatrist. The addition of case histories allowed for an interactive seminar. The attendees also received a printed copy of the materials reviewed	Two-day training for a total of 10 h, 1 month	Physician diagnosis from medical record and patient self-reported diagnosis using Zung Depression Scale and a symptom checklist that enabled making an approximate DSM-IV and ICD-10 diagnoses for a major depressive episode	The physicians diagnosed depression in 14.2% (*n* = 176) in phase 1 and 15.2% (*n* = 204) in phase 2 of the patients (change = 1%, *P* = 0.474). Agreement between the physician and patient self-reported diagnosis remained poor and showed no improvement following the educational program
Miller et al. (2020) [50]	A large perinatal collaborative care program was implemented. Educational programming pertaining to (1) the collaborative care model and (2) depression screening and treating was disseminated to obstetric providers and patients			Before intervention 129/1334 = 9.7% positive for depression and after intervention 310/3447 = 9.0%. Before intervention (postpartum care) 112/3097 = 3.6% positive for depression and after intervention 162/4167 = 3.9%

*WHO *World Health Organization, *mhGAP *Mental Health Gap Action Programme, *DSM* *Diagnostic and Statistical Manual of Mental Disorders*, *GPs *General practitioners, *HADS *Hospital Anxiety and Depression scale, *PCPS *Primary care providers, *PCDs *Primary care doctors, *CIDI *Composite International Diagnostic Interview, *SCID *Structured Clinical Interview for DSM-IV, *PRIME-MD *Primary Care Evaluation of Mental Disorders, *ICD *International Classification of Diseases, *GDS *Geriatric Depression Scale, *CES-D *Center for Epidemiologic Studies Depression Scale, *GHQ *General Health Questionnaire, *PHC *Primary healthcare, *OR *Odds ratio, *CEI *Canberra interview for the elderly

**Table 3:** Description of interventions and results of studies with screening and feedback intervention

Citation (author, year)	Description of the intervention	Duration of intervention, length of follow-up	Outcome measures	Estimate of effect/summary result
Davies et al. (2003) [51]	Health visitors used the EPDS routinely at 1, 3, 6, 12, and 18 months postpartum. If a score of between 9 and 11 was found, the health visitor immediately offered the opportunity for the woman to talk about her feelings and about any problems she was experiencing	Six months, 3 months, and 6 months	EPDS and patient records	Possible postnatal depression was detected at a rate of about 30% at the 1-month visit. This dropped to about 20% at the 3-month visit, 21% in 6 months, and 23% in 12 months
Kalina et al. (2016) [52]	Women were screened for postpartum depression using PHQ-2 at the 4- to 6-week and 6-month scheduled postpartum visits with the physician and nurse midwives. If a patient answered yes on one or more question on the PHQ-2, further screening using the PHQ-9 was performed	Three months, 6 months	PHQ-2	Before intervention 4/45 (8%) and after intervention 11/71 (15%)
Kozel et al. (2012) [53]	Each patient visiting the selected medical team at the outpatient clinic fills the Zung Self-Rating Depression Scale. The nurse evaluated the completed questionnaire and submitted the score to the family practitioner. After the examination, the family practitioner asked the patient two screening questions from PHQ-9. In the case that the first two answers to the screening questions were positive, the family physician asked the remaining seven questions	Three hours per day for 10 working days	PHQ-9 and Zung Self-Rating Depression Scale	Before pre-screening tool introduction: 5.7% After PHQ-9 introduction: 186/1930 = 9.6% After ZSRDS introduction: 209/1930 = 10.9% By the help of screening tools, family practitioners may diagnose more patients, who suffer from depressive disorder
Leslie et al. (2018) [54]	The provider was educated regarding the purpose, proper administration, and interpretation of the PHQ-9a. During the project, adolescents were requested to complete the PHQ-9a. Once completed, the provider reviewed the score and further evaluated and managed depressive symptoms as appropriate		PHQ-9	Before intervention: 15/282 = 5.3% and after intervention: 15/88 = 17%
Lewandowski et al. (2016) [55]	The Health Management Organization implemented the adolescent version of the Patient Health Questionnaire-9 (PHQ-9) as a screening tool in primary care. Previously, the PHQ-9 was used primarily in the mental health department for diagnostic support and monitoring of patients with a known diagnosis of depression		PHQ-9 and retrospective chart review	From 2010 to 2012, depression diagnosis increased by almost 40% in pediatric primary care and by almost 25% in adult primary care while decreasing by 13% in the mental health department
Libby et al. (2014) [56]	The PHQ-2 and PHQ-9 were embedded within the current electronic medical record as part of the template for 12 to 18 years old	8 weeks	PHQ-2 and PHQ-9	Before implementation, no diagnosis was recorded (diagnosis = 0%). After implementation, 12/264 = 4.5%
Leng et al. (2010) [57]	First patients categorized as language discordant (if the provider and patient spoke different languages) were randomized to RSMI or usual care interpreting. At each visit, research assistants facilitated patient receipt of their randomized method of interpretation, and medical charts were abstracted	18 months, 1 year	Beck Depression Inventory-Fast Screen and chart review	Depression diagnosis rate of 27% in RSMI group (intervention group) and 20% in usual care (control group), *P* = 0.41
Zupancic et al. (2010) [58]	Routine screening using Quick Inventory for Depressive Symptomatology Self-Report	Six months	Quick Inventory for Depressive Symptomatology Self-Report	Prevalence of depression pre-intervention was 38.9% (95/244), and during the intervention period, it was 54.8% (92/168) (*P* < 0.001)
Gledhill et al. (2003) [59]	The intervention consisted of three strands: (1) changing the consultation’s focus from presenting complaint (usually physical) to a psychological enquiry, (2) a diagnostic screen for depression based on standardized diagnostic criteria, and (3) treatment interventions adapted from cognitive-behavioral and inter-personal psychotherapeutic techniques	Two 1-h group sessions, 3 months	K-SADS psychiatric research interview and Mood and Feelings Questionnaire	Prior to training (GP+ = 6 and GP− = 32; K-SADS+ = 10 and K-SADS− = 28; sensitivity 20%, specificity 86%, and positive predictive value 33%) and after training (GP+ = 12 and GP− = 32; K-SADS+ = 21 and K-SADS− = 23; sensitivity 43%, specificity 87%, and positive predictive value 75%)
Badger et al. (1988) [60]	The GHQ was scored by the research assistant and affixed to the front of the patient’s chart prior to the medical examination; the residents, therefore, had access to the discrete items on the GHQ itself as well as to the sub-scores and summary score	Approximately 3 months, 3 months	Chart audit form: psychiatric diagnosis was derived for each resident from the assessment portion of the problem-oriented SOAP note	Pretest: (control = 12 vs experimental = 5), *P* < 0.02; posttest (control = 5 vs experimental = 25), *P* < 0.02
Callahan et al. (1996) [61]	The GHQ was scored by the research assistant and affixed to the front of the patient’s chart prior to the medical examination. The residents, therefore, had access to the discrete items on the GHQ itself as well as to the sub-scores and summary score	One year, 1 year	A structured questionnaire to describe physicians’ clinical assessments of elderly patients and patients’ encounters	Patients with specific chart documentation of depressive symptoms (intervention = 86.7% and control 40.4%, *P* = 0.001) and symptoms of major depression (intervention = 50.8% and control = 39.4%, *P* = 0.09)
Christensen et al. (2003) [62]	All patients were screened in the waiting room before consultation. A one-page screening questionnaire was used, consisting of various rating scales. After completion, patients were randomized to either blinding or disclosure of the screening questionnaire information to the GP	3-week period (3 March–1 May 2000), 3 weeks	The GPs were specifically asked whether the patient suffered from a significant psychiatric disorder and a semi-structured standardized psychiatric interview	GP sensitivity was 50.0 (36.7 to 63.3) versus 40.6 (31.0 to 51.0), GP specificity was 95.0 (91.0 to 97.2) versus 94.7 (89.4 to 97.4), and correctly classified was 89.1 (84.4 to 92.5) versus 87.3 (82.6 to 90.9) in the disclosure and blinding
German et al. (1987) [63]	The GHQ results were given to the clinicians before or at the same time they saw the patient. The feedback form gave the total score, the subscale scores, the individual items the patient had answered positively, and an explanation of the GHQ	Not stated, 6 months	Short instrument filled out by the clinician at the time of the visit. The clinicians completed a clinician report form	Age < 65 (total = 343/57.7%; no feedback = 223/57.4%; feedback = 120/58.3%) age > 65 (total = 133/48.1%; no feedback = 92/41.3%; feedback = 41/63.4%)
Moore et al. (1978) [64]	A note was attached to the patient’s visit form indicated to the physician, before seeing the patient, that this individual scored in the “ mildly depressed” (SDS between 50 and 60) or “severely depressed” (SDS greater than 60) range. For patients with SDS scores less than 50, residents also received a card indicating only that the patient had been screened	Two months	Kutcher Adolescent Depression Scale and chart review	When alerted, *SDS* > 50, 28/50 = 56% noted depression diagnosis. When alerted, *SDS* > 60 16/41 = 72.7% noted depression diagnosis
Rand et al. (1988) [65]	Residents in the experimental site were introduced to the GHQ and were trained to interpret section scores as well as total GHQ scores. The GHQ was scored by the research assistant and affixed to the front of the patient’s chart prior to the medical examination; therefore, the residents had access to the discrete items on the GHQ itself as well as to the sub-scores and summary score	Three months	The 28-item GHQ and PHC staff chart note	Control site residents made a total of 38 diagnosis as compared to the experimental site total of 19 (*P* = 0.02). Post intervention, the experimental site residents increased the diagnoses from 19 to 53 compared to 34 to 30 in control site. Number of patients with a diagnosis of depression increased from 5 to 25 in experimental group
Schriger et al. (2001) [66]	Physicians caring for patients in the report group were provided with the results of the computer interview. The 2-page report contained the output of the PRIME-MD software. This report was paper clipped to the front of the physician section of the medical record	Not reported	Computer version of PRIME-MD (software) was used to screen patients for psychiatric disorder	PRIME-MD made at least 1 psychiatric diagnosis in 46% of control group patients and 37% of report group subjects (difference 9%, 95% *CI* = −5%, 23%). Major depression was diagnosed in 16% of control group patients and 16% of report group subjects

*EPDS *Edinburgh Postnatal Depression Scale, *PHQ *Patient Health Questionnaire, *ZSRDS *Zung Self-Rating Depression Scale, *GP* General practitioner, *GHQ *General Health Questionnaire, *SDS *Self-rating Depression Scale, *PRIME-MD *Primary Care Evaluation of Mental Disorders

**Table 4:** Description of interventions and results of studies with combination of interventions (education, screening, and feedback)

Citation (author, year)	Description of the intervention	Duration of intervention, length of follow-up	Outcome measures	Estimate of effect/summary result
Albedaiwi et al. (2005) [67]	Three-part intervention (raising physicians’ awareness of depression, mass depression screening using a 2-item version of the Prime-MD Questionnaire, and communicating the results of the screening to the physician)	Five months, 3 months	Identifying patients already receiving antidepressant treatment and identifying those started on treatment for depression at the time of the screening visit	A total of 10.9% pre-intervention and 16.3% post-intervention. This approach (the intervention) can greatly enhance the recognition of depression in primary care patients
Diez-Canseco et al. (2018) [68]	The intervention combined three strategies: training of PHC providers, task shifting the detection and referral of mental disorders, and a mobile health (mHealth) component comprising a screening app followed by motivational and reminder short message service to identify at-risk patients	Nine weeks	Follow-up interviews with patients and midterm and post-intervention interviews with PHCPs	Out of the 733 patients screened, 159 of them (21.7%) had a positive result by the SRQ. Of those 159, 150 (94.3%) screened positive for one disorder and 9 (5.7%) for two disorders. The average score at pretest was 12.1 (*SD* = 2.3) and 16.5 (*SD* = 1.35) at posttest
Linn et al. (1980) [69]	Group 1 completed the Zung Self-Rating Depression Scale (SDS). The scale was scored, photocopied, and attached to the patient’s chart with a note. The physician was given a picture of a 10-rung ladder to rate how his patient was feeling. Group 2 patients were asked to complete the SDS, but results were not given to the physician until after the encounter was completed. The physician first asked to rate the patient’s level of depression by use of the ladder, and then, the doctor was given feedback from the SDS screening. Group 3 patients completed the SDS, and the results were given to the physician before the encounter, but the physician’s opinion about the patient’s level of depression was not requested. Group 4 patients were asked to complete the SDS, but results were not given to the physician until after the encounter was terminated. The physician’s opinion about the patient’s level of depression was not requested. Group 5 patients were not screened for depression; however, the physicians were asked to evaluate the patient’s level of depression. Group 6 patients were not screened for depression, and the physicians were not asked to evaluate depression	Single visit, 2 weeks	Review of medical records for notations regarding depression and its treatment	When groups 1 through 4 are combined (patients receiving screening and physicians receiving feedback) and compared with groups 5 and 6 combined (no screening or feedback), notation of depression was three times more likely (25% versus 8%, *P* < 0.01). When groups 1 and 2 are considered together and compared with all others, the combination of screening, feedback and sensitization resulted in significantly more frequent notations of depression (32%) than any single intervention or none (13%, *P* < 0.01). No significant differences in notation were found when all sensitization groups (1, 2, and 5) were compared with none-sensitized groups (3, 4, and 6) (24% versus 14%)
Romera et al. (2013) [70]	PCPs in the intervention arm received a 1-day face-to-face training by a psychiatrist and four PCPs on the recommendations on screening for depression in adults, according to USPSTF 2002 guidelines, and were asked to implement them in their routine clinical practice for at least 6 months. Monthly reminders were sent by e-mail. They were also required to complete a form each month indicating adherence and acceptance and feasibility	Six months, 6 months	Systematic review of the patients’ medical records carried out by the participating physicians, Hospital Anxiety and Depression Scale, and the Mini-International Neuropsychiatric Interview	Under-recognition rates were 33.9% in the intervention and 41.4% in the control. Recognition rates were 58.0% in the intervention and 48.1% in the control: *OR* [95% *CI*] 1.40 [0.73–2.68], *P* = 0.309)
Sorkin et al. (2019) [71]	Multicomponent mental health-IT intervention: (I) medical providers completed an online tutorial on how to provide culturally competent, trauma-informed mental health care, (ii) screening all patients just before their appointment with their physician using 2 culturally adapted instruments for depression and PTSD and providers received a printed notification results, and (iii) giving medical provider access to evidence-based clinical algorithms and guidelines through a web-based mobile application	The web-based tutorial is -h training, and duration of other components is not clear, 12 weeks	Depression was assessed using the 15-item depression subscale of the Hopkins Symptom Checklist	Approximately 81% was diagnosed with depression by their providers in the treatment group, whereas only 33% was diagnosed by their providers in the control group. Providers in the intervention were more likely to diagnose depression [odds ratio (OR), 6.5; 95% confidence interval (CI), 1.48–28.79; *P* = 0.013]
Whooley et al. (2000) [72]	PCPs in both intervention and control clinics were provided an educational session to improve their management skills of depression. PCPs in the intervention clinics were notified of each participant’s GDS score and given an instruction sheet indicating the ranges of scores associated with depression	One-hour educational session for physicians and for patients weekly educational sessions, 2 years	Blinded review of all outpatient physician diagnosis forms, telephone interview, and GDS	Fourteen percent of participants (331/2346) had GDS scores suggestive of depression (GDS ≥ 6) at baseline. During the 2-year follow-up period, 56 (35%) of the intervention participants and 58 (34%) of the control participants received a physician diagnosis of depression
Williams et al. (1999) [73]	Physicians were given a copy of the Quick Reference Guide for Clinicians on managing depression in primary care and a continuing medical education session on interpreting case-finding questionnaires and diagnosing and treating depression. Patients assigned to case finding completed the depression questionnaire, and the results were reported to their physician on a bright orange report form. Questionnaire results were reported as negative or positive	Three months	Recognition rates of depression were assessed from medical records using chart audits and compared against DSM-III diagnosis	The intervention modestly increased depression recognition (30/77 or 39%) compared with 11/38 or 29% in usual care); difference = 10%, *P* = 0.31) but did not affect treatment (45% vs. 43%, *P* = 0.88)
Yonkers et al. (2009) [74]	The program included provider education, community awareness, and direct systematic depression screening. Educational and training seminars were run for healthcare clinicians and NHHS care coordinators. Women enrolling in NHHS were administered a standardized risk assessment that included questions regarding general medical and obstetrical problems, PRIME-MD, and Brief Patient Health Questionnaire	Three years, 10 months	Detection was determined with women’s self-report and chart review	Rates of detection by the obstetrical provider differed significantly between the three groups (15%, 6%, and 5% for groups 1, 2, and 3, respectively) (*P* = 0.003)
Yawn et al. (2012) [75]	A practice-wide intervention consisting of postpartum depression screening, evaluation, and management based in family medicine offices. Intervention staff members received training for a multistep postpartum depression screening and diagnosis process using the EPDS and PHQ-9. They also received training and practice of nursing telephone calls and on scoring and using PHQ-9. The intervention sites had routine access to the woman’s EPDS and PHQ-9 screening score(s)	Half-day training (2 sessions 1 h each) and refresher session 6 weeks later. Control sites were given a 1-h program, 12 months	EPDS, PHQ-9, and the Structured Clinical Interview for the *Diagnostic and Statistical Manual of Mental Disorders*	Three-hundred thirty-nine (29.5%) of the intervention women and 255 (25.8%) of the usual-care women reported depression symptoms. Diagnosis for postpartum depression was significantly more likely among the intervention women. That is, among those with elevated screening scores, 78/399 (41%) of usual care and 194/255 (66%) intervention receive a diagnosis (*P* = 0.0001)

*PRIME-MD *Primary Care Evaluation of Mental Disorders, *PHC *Primary healthcare, *PHCPs *Primary healthcare providers, *SDS *Zung Self-Rating Depression Scale, *CI *Confidence interval, *OR *Odds ratio, *PTSD *Post-traumatic stress disorder, *GDS *Geriatric Depression Scale, *DSM* *Diagnostic and Statistical Manual of Mental Disorders*, *EPDS *Edinburgh Postnatal Depression Scale, *PHQ *Patient Health Questionnaire

**Table 5:** Description of interventions and results of studies with development and dissemination of guidelines/collaborative care package/quality improvement interventions

Citation (author, year)	Description of the intervention	Duration of intervention, length of follow-up	Outcome measures	Estimate of effect/summary result
Bermejo et al. (2007) [76]	Evidence-based guideline training on the identification of depressive syndromes by GPs designed as a multifaceted, interdisciplinary intervention, combining benchmarking, interactive continuous medical education, and interdisciplinary quality circles	Six sessions, each of 3 h within a 10-month period, posttest at the end of the intervention and follow-up at 12 months	The German depression module of the Patient Health Questionnaire. For each patient, the physicians evaluated whether the patient had a depressive disorder	In the follow-up assessment, the concordance rate increased from 51.2 to 57.1%. • Intervention group sensitivity: 26.4% vs 51.1% vs 56.2% • Control group sensitivity: 47.2% vs 54.5% • Intervention group specificity: 87% vs 87.3% vs 80.9% • Control group specificity: 81.5% vs 81.9%
Croudace et al. (2003) [77]	Local development and dissemination of the WHO ICD-10 PHC guidelines (1996 version, which was “current” at that time). Participating GPs were provided with the opportunity to adapt the WHO guidelines in a shared-ownership model with colleagues from local psychiatric services. Participating GPs received a personal, desktop copy of the guidelines. Educational meetings were then organized in each intervention practice	One year, 1 year for practice and GP level outcomes and 3 months for patient-level clinical outcome	A GHQ–12 score of > 3 was used to define a case. Functioning was recorded using the Brief Disability Questionnaire. Quality of life was recorded by the European Quality of Life instrument. A single question assessed satisfaction with care	Detection rate (sensitivity) for GPs in the guideline practices was 47% and 55% in the usual care. After adjustment for baseline sensitivity, the difference was 76.6% (95% *CI* 719.0 to 5.9%; *z* = 1.03%, *P* = 0.304). The crude specificities achieved by guideline and usual-care practices were 86% and 79%, respectively. After adjustment for baseline specificity, this difference increased slightly to 6.2% (95% *CI* 74.4 to 16.8%; *z* = 1.14, *P* = 0.255)
Dwinnells et al. (2015) [78]	Behavioral health Screening, Brief Intervention, and Referral to Treatment (SBIRT) program: Before SBIRT implementation, the medical and clerical staff received 3 educational sessions to learn about the SBIRT. Patients were screened for depression, alcohol, and substance use. Those eliciting positive responses were given appropriate, quantifiable standardized tests	Six months, 6 months	The screening tool, consisting of 5 dichotomous questions adapted from the Oregon Health & Science University’s SBIRT program; standardized tests (e.g., PHQ-9)	Compared with 11.4% of the control site patients, 25.3% of the SBIRT intervention site patients had positive findings for depression, alcohol, or substance use (*P* < .001)
Jordans et al. (2019) [79]	A Mental Health Care Plan (MHCP) developed and implemented in Nepal, in partnership with the Ministry of Health. Comprised interventions at the community, health facility, and health service organization levels. Packages included training and supervision for health workers to detect, diagnose, and initiate treatment for a priority disorder (i.e., depression, psychosis, AUD, and epilepsy)	Cross-sectional surveys with independent sampling were conducted before MHCP implementation and approximately 6 months and 24 months after initiating the MHCP	PHQ-9	At baseline, 186/1252 were positive (15%); of those 186, 179 seen by professionals and 8.9% diagnosed as having depression. At post intervention increased to 24.6% after 6 months (*ES* = 0.432) and at 24 months 19.2% (*ES* = 0.301)
Nakku et al. (2019) [80]	MHCP and mhGAP implementation	Twelve months, every 3onths for a year	PHQ-9	At baseline, 325/1290 were PHQ-9 positive of which 85 were new (85/1290 = 6.7%), and at end line, 452/3481 (12.9%) were PHQ-9 positive. The improvement in detection of depression at 3 months was not sustained over 12 months
Petersen et al. (2019) [81]	Comprised five components: (i) PHC nurses functioned as case managers, oriented to the ICSM, trained in communication skills, and provided with mental health training, (ii) doctors were oriented to the importance of mental health and upskilled to prescribe antidepressants, (iii) referral pathways for psychosocial counseling were strengthened, and (v) a referral form to monitor nurse referrals to the counselor was introduced	Twelve months	PHQ-9 (with a cutoff of ≥ 10) detection by clinicians was assessed by asking screen positives	Using the narrow definition, detection of depressive symptoms increased from 5.2% (6/102) to 16.2% (19/116). Using the broad definition, detection of depressive symptoms increased from 14.2% (14/102) to 26.7% (31/116)
Sherman et al. (2004) [82]	Evidence-based quality improvement intervention: The intervention development process consisted of four steps: setting of priorities for managing depression, expert adaptation of the priorities, development of the QI Plan, and implementation of the QI Plan	Ten months	Ten-item questionnaire and 2-item questionnaire from PRIME-MD to screen patients for depression. Detection of depression assessed by looking at the action they took in the medical chart or notes	At pre-implementation, 97/264 (37%) were positive for depressive symptoms, and only 11 (30%) were recognized by the provider as having depressive symptoms. At post-implementation, 90% of the patients were screened, and of them, 20–25% were screened as positive
Scott et al. (2002) [83]	A “chronic disease management” approach: The intervention was multifaceted, including resources to develop a case register, training program on detection, facilitation of meetings with secondary care staff, and support in developing a practice guideline	Not stated	Hospital Anxiety and Depression Scale and GPs diagnosis	At practice A, sensitivity improved by 23% (from 60 to 83%), and specificity decreased from 62 to 60%. At practice B, sensitivity increased by 25% (25 to 50%), and specificity decreased from 94 to 72%
Upton et al. (1999) [84]	Introduction of ICD-10 guideline which consisted of a study day and the provision of a book of guidelines for mental disorders. The guidelines consist of a brief description of the disorder, a list of diagnostic features, and cross-references to differential diagnoses. The management guidelines consist of information for the patient and family, medical, psychological and social interventions, and referral criteria. The study day consisted of an overview of mental disorders in PHC, the development of ICD-10 PHC, and role plays	Eleven weeks before and 11 weeks after the introduction of the guidelines	GP diagnosis, GHQ-12, and clinical interview schedule	Prevalence of depression diagnosis before was 238 per 10,000 consultations and after 305 per 10,000 (difference = 0.7%, 95% *CI* 0.2–1.1). Kappa (95% CI) before vs. after was 0.31 (0.1–0.52) fair vs. 0.11 (0–0.34) slight. Of those scoring 2 or more in GHQ12, the GPs identified about 51% before and 54% after as having a mental health disorder
Rinke et al. (2019) [85]	Quality improvement collaborative which is an organized, multifaceted collaborative approach to QI with (1) a specific topic for improvement with large practice variation; (2) clinical and QI experts sharing best practices; (3) multidisciplinary teams from multiple sites willing to improve; (4) a model for improvement with measurable targets, data feedback, and small tests of change; and (5) a series of structured activities to advance improvement, exchange ideas, and share experiences	Two-day interactive video learning session plus monthly video conferences, 8 months	PHQ-9 modified and clinical judgment	The adjusted percentage of patients with depression, dysthymia, or subsyndromal depression diagnoses is 6.6% in the control phase and 10.5% in intervention phase (risk difference (RD) 3.9%; *P* < 0.0001). Practices sustained these increases during the second (*RD* −0.4%, *P* = 0.642) and third action periods (*RD* −0.1%, *P* = 0.911)

*GPs *General practitioners, *WHO *World Health Organization, *ICD *International Classification of Diseases, *PHC *Primary healthcare, *GHQ *General Health Questionnaire, *CI *Confidence interval, *SBIRT *Behavioral health screening, brief intervention, and referral to treatment, *PHQ *Patient health questionnaire, *ES *Effect size, *MHCP *Mental health action plan, *mhGAP *Mental Health Gap Action Programme, *QI *Quality improvement, *PRIME-MD *Primary Care Evaluation of Mental Disorders, *RD R*isk difference

Most of the clinician training interventions involved didactic sessions on the diagnosis, treatment, and referral of PHC depression [31, 32, 34, 37, 47]. The use of active learning methods (discussion, role play, and presentation) was particularly useful [35, 42, 45]. Training interventions included a theoretical part and a practical part, focusing on case discussion, role-playing games, and use of vignettes for clinical cases [30, 36]. Many of the educational interventions also involved clinical practice in assessment and treatment of depression using real or standardized patients or visit to outpatient departments [35–37, 40, 42]. A few educational interventions involved providing educational packet which included a copy of screening tool, treatment algorithm, and medication dosing guidelines [24, 41]. Training on the use (administration, scoring, and interpretation) of a depression screening scale was also an important aspect of educational interventions [49, 50]. One study tested a tailored and activating educational intervention (tailored according to the participants’ readiness to change) [48]. The duration of the training was diverse, ranging from 1 h to 2 weeks [31, 36, 45], and trainers were mostly psychiatrists or clinical psychologists. See Table 2.

Regarding studies which evaluated screening/feedback interventions, several types of screening tools were used, including different versions of the Patient Health Questionnaire (PHQ) [52–56], Zung Self-Rating Depression Scale (SDS) [53, 64], Quick Inventory for Depression Symptomatology Self-report (QIDS-SR) [58], General Health Questionnaire (GHQ) [60, 61, 63, 65], Edinburgh Postnatal Depression Scale (EPDS) [51], and Primary Care Evaluation of Mental Disorders (PRIME-MD) [66] (Table 3). Nevertheless, PHQ and GHQ were the most commonly used screening tools. The screening tools were mostly administered by nurses, general practitioners, and research assistants. In some studies, screening tools were self-administered [53, 58], and scoring was done by either nurse assistants or healthcare providers. While some of the studies used screening only as intervention [51, 52, 55–59], other studies used screening with feedback [53, 54, 60–66]. In most cases, results were given to the clinicians before or at the same time they saw the patient. The feedback gave the total score, the subscale scores, and the individual items the patient had answered positively.

Taking into account the limitations and strengths of each of the three types of interventions (clinician training, screening, and communicating the results of screening to clinicians), some studies combined two or all the three types of these interventions (Table 4). While some of the studies combined providing brief training to clinicians and screening of depression [68, 70, 74, 75], others combined all the three types of interventions: providing training to healthcare providers, depression screening with brief structured tools, and then providing feedback to the providers about the results of the screening [67, 71–73]. One study combined screening and then communicating screening results to clinicians [69].

The last type of intervention was related to development or adaptation and implementation of evidence-based guidelines, collaborative care packages, and quality improvement programs. This type of intervention was diverse in terms of who developed the intervention, its content, and duration of implementation (Table 5). Some examples of this type of intervention included implementation of evidence-based guideline on the identification of depressive symptoms [76], the World Health Organization’s International Classification of Diseases 10th version (WHO ICD-10) PHC guideline [77, 84], Behavioral health Screening, Brief Intervention, and Referral to Treatment (SBIRT) program [78], Mental Healthcare Plan (MHCP) [79]; adaptation and implementation of the WHO mhGAP [80]; implementation of a chronic diseases management approach [83], and organized, multi-faced collaborative approach to quality improvement [82, 85]. The guidelines/care packages/quality improvement programs were either locally developed by the research team or adapted from international guidelines/care packages/quality improvement programs. The duration of implementation varied from as short as 3 months [84] to as long as 12 months [77, 80, 81].

### Effectiveness of interventions to improve detection of depression in primary healthcare

Of the 58 studies included in the review, 42 found positive results (i.e., significant improvement in the detection of depression in the PHC setting). Interventions that were found to be usually effective included implementation of comprehensive evidence-based guidelines/collaborative care packages/quality improvement programs [78, 79, 81, 83, 85]; screening with feedback supported by basic training and supervision [67, 74, 75]; multifaceted educational interventions which incorporated active learning, discussion, role play and clinical practice [33, 35, 36, 40, 48];and feedback or disclosure of screening results, particularly, of high screening scores [60, 61, 64, 65]. Interventions which combined education, screening, and feedback were particularly more effective in increasing recognition of depression [67, 74, 75]. Training interventions usually had short-term effects. Improvements in detection of depression after training of healthcare staff were observed; however, this improvement was not sustained.

### Effectiveness of clinician training/education interventions

A study which evaluated a single evening 3 and half hour long seminar, which focused on the diagnosis, treatment, and referral of depression brought about significant improvement in accuracy of depression diagnosis (mean depression diagnosis accuracy in the intervention group 1.35 and in the control group 0.97) [32]. Another study, which used a 2–3 training sessions together with regular consultations between GPs and psychiatrists increased cases with clinically diagnosed depression from 4.0 to 7.9% (*P* < 0.05) [33]. Agreement between HAD diagnosis and clinical evaluation of depressive disorders improved from 20% (*k* = 0.18) to 45% (*k* = 0.54). After 1 year, GPs identified twice as many of the patients that suffered from anxiety or depression in comparison to before the intervention. A training intervention which involved a 60-min seminar plus a 60 min practical clinical skill improved diagnosis of depression among adolescents (from 0.89 to 2.22%) [35]. The odds of receiving a new diagnosis of depression were almost three times higher after training (*OR* = 2.7; *P* < .0001)

Two weeks onsite training which consisted of 2 h of didactic session on a topic, followed by a 2 hour visit to the outpatient department significantly improved mean scores on case vignettes (from 42.4 at baseline to 83.4 post-training) [36]. Eight hours training on depression to PHC providers, which consisted of a theoretical part and a practical part focusing on case discussion, role play, and use of case vignettes increased the rate of identification of depression from 5.9% (*n* = 97/1647) before training to 10.64% (*n* = 196/1832) after training [37]. Five-day training on mental health using a toolkit originally designed for Kenya improved detection of depression from 0 to 9% [40]. A 1-h minimal education intervention consisting of information, skill training, and discussion in small groups significantly improved recognition of depression among the elderly 3 months following the intervention (25% in the intervention group vs. 7% in the control group) [45]. One study tailored the intervention according to readiness to change and used interactive and multi-faceted learning activities, including case illustrations, standardized patients, role play, buzz group and programmed lecture [48]. The intervention was 2-day interactive training workshop. Printed materials were also given to the training participants. At post-intervention, mean of performance regarding depression diagnosis at the intervention group A vs. control group was 63 and 49, respectively (*P* = 0.007), whereas intervention group B vs. control group was 49 and 22, respectively (*P* < 0.001).

A study which evaluated a 3-day training course locally developed from the WHO mhGAP intervention guide in Nigeria on ability of PHC workers to make accurate diagnosis of depression found no improvement (92.5% pre-training and 93.8% post-training, *P* = 0.200) [30]. One hour training which provided basic information about depression and demonstration of a depression screening strategy did not improve accuracy of identification of cases of depression (22.2% in the intervention group and 16.7% in the control group, *P* > 0.05) [31]. Participants who were given a standard 2 h training which had role play did not bring improvement in their ability to recognize depression (1.91 per 100 visits in the intervention and 1.68 in the control were identified) [42]. A study tested the effectiveness of a brief 2-day training program for depression designed by the World Psychiatric Association (WPA) using a training module in the form of a seminar [49]. Case histories were used to make the seminar interactive. The attendees also received a printed copy of the materials. The participating physicians diagnosed depression in 14.2% (*n* = 176) of the patients before the training and 15.2% (*n* = 204) after the training (change = 1%, *P* = 0.474). Agreement between the physician and patient self-reported diagnosis remained poor and showed no improvement following the educational program.

A study tested the effect of a training which consisted of eight sessions of 2.5 h each [46]. Each session followed a similar structure: discussion of the normal practices and difficulties of the trainees; a short lecture by the psychiatrist trainer; illustration with video-taped consultations; introduction of guidelines and protocols for screening, diagnosis, or interventions; practice using various forms of hands-on learning (e.g., role playing); and evaluation. The study found no overall significant pre-post differences (*OR* = 1.39, *P* = 0.15) in diagnosis of depression. Rate of depression diagnosis was pre-training 40% and post-training 48%, *P* = 0.12).

### Effectiveness of screening interventions with or without feedback

Overall, implementing routine screening of depression in the PHC setting improved detection of depression [51, 52, 54, 56]. These improvements did not mostly have statistical and practical significance. Almost all of the studies which tested screening intervention, however, had before-after quasi-experimental design. Implementation of routine screening of depression in the PHC setting was found to be more effective in terms of increasing recognition of depression when it was accompanied by feedback (disclosure of screening results to clinicians). Almost all of the studies, with screening intervention accompanied by feedback, brought about significant improvement in detection of depression [60, 61, 64, 65]. Nevertheless, many of the studies were non-randomized trials.

### Effectiveness of combined education, screening, and feedback interventions

Improvement in PHC diagnosis of depression was found to be much higher when the three types of interventions were combined (clinician training, screening, and feedback). Almost all of the studies which evaluated a combination of these interventions (e.g., raising clinicians’ awareness and skills of managing depression, mass depression screening, and communicating the results to the clinician) found significant increase in recognition of depression [67, 69–71, 73–75]. A study which evaluated combining screening, feedback, and sensitization (by asking the clinician to rate the patient’s level of depression) improved detection of depression (32% in the intervention group compared to 8% in the control group) [69]. A few studies also evaluated interventions which combined clinician training and screening [68, 70, 71, 74]. Although these studies found improvement in PHC diagnosis of depression, effects were not as much as those interventions which combined education, screening, and feedback. Two out of the four studies (which tested interventions combining education and screening) did not find statistically significant improvement in detection of depression [68, 70].

### Effectiveness of implementation of guidelines/collaborative care packages/quality improvement programs

Six of the ten studies [78–81, 83, 85], which evaluated effectiveness of implementation of guidelines/collaborative care packages/quality improvement programs found significant improvement in detection of depression at the PHC setting. For instance, implementation of a behavioral health Screening, Brief Intervention, and Referral to Treatment (SBIRT) program [78] found that 25.3% of the SBIRT intervention site patients had positive findings for depression compared with 11.4% of the control site patients (*P* < .001). A Mental Health Care Plan (MHCP) developed and implemented in Nepal, with training packages and supervision for health workers to detect, diagnose, and initiate treatment for depression increased the recognition of depression 15 to 24.6% [79]. Four of the ten studies, on the other hand, did not find statistically significant improvement [76, 77, 82, 84]. For instance, local development and dissemination of the WHO ICD-10 PHC guidelines [84] found no significant difference between guideline practices and usual-care practices. These interventions were diverse and multi-faceted, which involved development or adaptation and implementation of guidelines/care packages/quality improvement programs, supervision, collaboration and training.

### Quality of included studies

Assessment of the quality of the included articles using the Quality Assessment Tool for Quantitative Studies showed weak quality in 14 studies out of 58. More than half of the studies (30 out of 58 articles) were rated as moderate quality, and the remaining 14 were rated as strong. In terms of selection bias, 31 studies were rated as moderate, 14 as weak, and 13 as strong. Most of the studies were rated as moderate (20 out of 58 studies) or strong (21 out of 58 studies) in their study design; 17 studies were rated as weak. Most of the studies were rated as weak (17 out of 58 studies) or moderate (30 out of 58 studies) in terms of blinding. Only 11 studies were rated as strong quality in taking care of blinding. More than half of the studies were rated as strong (35 out of 58 studies) in adjusting for confounding factors, whereas 18 studies were rated as weak and five studies as moderate. With regard to withdrawal and dropout, 24 out of 58 studies were rated as weak, 13 studies as moderate and 21 studies as strong. For details of the results of the quality assessment, see Additional file 2.

## Discussion

To our knowledge, this is the first systematic review study which synthesized the global evidence on the effectiveness of diverse types of interventions to improve the detection of depression in the primary healthcare setting. Previous systematic reviews focused on effectiveness of single interventions, such as clinician education [22] and routine screening [16]. The current review, on the other hand, included studies which tested the effect of diverse interventions to improve primary care clinicians’ diagnosis of depression. Most of the studies are conducted in high-income countries; only six of the 58 studies were from low-income countries. Most of the studies (44 out of 58 studies) are weak or moderate in their global rating of quality assessment; only 14 studies are found to have strong methodological quality. This is on the basis of the results of the assessment of the quality of the included studies using the “Quality Assessment Tool for Quantitative Studies.”

Six groups of interventions were identified: clinician education, screening, screening with feedback, combination of interventions, and implementation of guidelines/collaborative care packages/quality improvement programs with one study testing the effect of patient request for antidepressants. Different studies evaluated the effectiveness of the same intervention (e.g., clinician education, screening or feedback); however, the duration, intensity, content, and format of the interventions in each study are quite different.

The studies included in the review are heterogeneous in terms of the type of intervention and how they delivered the same type of intervention making it difficult to assess the comparative effectiveness of the different interventions. The studies were also heterogeneous in their design, follow-up period, outcome measure, and the method of data analysis they used. The heterogeneity of the included studies in the review, particularly in terms of study design, is partly due to the broader nature of our inclusion criteria. Nevertheless, there were aspects of the different types of interventions that seemed particularly effective. These included screening with feedback and combining training, screening, and feedback interventions. Training interventions showed some improvement in the detection of depression in PHC. However, effects are short term and do not sustain after 6 months. A previous systematic review on the effectiveness of capacity building or training of primary healthcare professionals in the detection of depression in PHC found consistent results with our review [22]. Training interventions which used active learning methods, including role play, discussion, and clinical practice, are particularly more effective than those which used didactic sessions. There seems consensus that education and training will be more effective when it is participatory and active. The knowledge gained and the skills developed will also be long lasting when a training program uses active learning methods such as role play, presentation, and discussion. When didactic sessions dominate a training/education program, then skills will not be developed, and the knowledge obtained will be short lived. Local adaptation and implementation of guidelines, collaborative care packages, and quality improvement programs are also mostly effective. These interventions seem resource intensive; however, they are cost-effective in the long term [86].

Most of the included studies were conducted in high-income countries, with just six of the 58 studies carried out in low-income country settings. There is a large difference, both in the rate of detection and treatment coverage of depression, between high-income countries and LMICs [87]. Thus, findings from high-income countries cannot be generalized into LMICs. The methodological quality of most studies was also rated as weak or moderate necessitating new high-quality studies with randomized-controlled trial designs, particularly in LMIC settings.

Our review indicates that combination of interventions is likely to be required to improve the detection of depression in a PHC setting. There is need to focus on combining interventions and implement them in the form of guideline, collaborative care package, or quality improvement program. Implementation of routine screening alone does not seem to be effective; it has to be accompanied by disclosure of screening results to the clinician, training of the clinical and other relevant staff, and supportive supervision. A previous systematic review that was done to determine the effect of screening on improving recognition of depression [16] in the PHC setting found that if used alone, it appears to have little or no impact. This finding is on both the detection and the management of depression by PHC clinicians.

Our review also indicates that the implementation of guidelines, collaborative care, and quality improvement programs are more effective than usual care both for increasing the detection rate of depression in PHC and improving outcomes of depression treatment. A previous systematic review and meta-analysis of effectiveness of collaborative care for depression [86] found that collaborative care packages significantly improve recognition of depression by PHC workers. This intervention also improved outcomes of depression treatment in the PHC setting.

Little research has been done in LMICs, despite the rate of detection and treatment coverage of depression in PHC being extremely low [87]. Hence, more number of randomized-controlled trials, which addressed the limitations of previous studies and focused on more effective interventions, need to be conducted. There is also uncertainty about the sustainability of the effect of the interventions, which should be addressed in future studies.

Our systematic review is comprehensive in that we searched more than five databases with detailed search strategy and no geographical restriction. In addition, we consulted the references of the included articles. Our review covered studies which tested all types of interventions to improve the detection of depression in PHC. However, the findings of this review should be interpreted taking the following limitations into account. Our systematic review included studies which are published only in English language. Our review also did not search the gray literature. The studies included in the review were heterogeneous in terms of design, type, and content of the intervention, outcome measure, and measure of effect. Even when different studies evaluated the same intervention, they used different designs, different methods of analysis, and also operationalized detection of depression quite differently. Hence, it was not possible to conduct meta-analysis and network meta-analysis. Most of the included studies were from high-income countries which make it difficult to generalize the findings into low-income country settings. Most of the studies were weak or moderate in their methodological quality; about one in every four studies was rated as weak.

## Conclusions

Our systematic review shows that several types of interventions were tested to improve the rate of detection of depression in PHC. Nevertheless, findings are inconsistent, and implementation of a single type of intervention does not seem to work. Implementation of a combination of interventions, focusing on aspects of each type of intervention which are more effective, seems promising. Combining healthcare staff training, screening, and feedback interventions is likely to increase recognition of depression in LMICs. Clinician education and training interventions, which incorporated simulation and role play, are found to be more effective. There is need to evaluate effectiveness of implementation of collaborative care packages and quality improvement programs to improve detection of depression in PHC in low-income country settings. Most of the studies conducted in the area are from high-income countries; studies are also weak in terms of their methodological quality. Hence, conducting high-quality RCT studies particularly from LMICs is warranted.

## Supplementary Information

**Additional file 1.** 

**Additional file 2.** 

**Additional file 3.**

## Data Availability

All of the data used for the study are made available within the manuscript and supplementary materials.

## Abbreviations

AIM: African Index Medicus
AJOL: African Journals OnLine
DALYs: Disability-adjusted life years
EPDS: Edinburgh Postnatal Depression Scale
EPHPP: The Effective Public Health Practice Project
GHQ: General Health Questionnaire
ICD-10: International Classification of Diseases 10th version
LILACS: Latin American & Caribbean Health Sciences Literature
LMICs: Low- and middle-income countries
MHCP: Mental Healthcare Plan
mhGAP: Mental Health Gap Action Programme
OR: Odds ratio
PHC: Primary healthcare
PHQ: Patient Health Questionnaire
PRIME-MD: Primary Care Evaluation of Mental Disorders
PRISMA: The Preferred Reporting Items for Systematic Reviews and Meta-Analysis
RCT: Randomized controlled trials
QIDS-SR: Quick Inventory for Depression Symptomatology Self-report
SBIRT: Behavioral health Screening, Brief Intervention, and Referral to Treatment
SDS: Zung Self-Rating Depression Scale
WHO: World Health Organization
WPA: World Psychiatric Association
