# Supplementary material for: Interventions to improve the detection of depression in primary healthcare: systematic review
Source: Syst Rev. 2023 Feb 24;12:25. doi: 10.1186/s13643-023-02177-6 (PMC9951508; doi:10.1186/s13643-023-02177-6)
Supplement: Supplementary file 1 — Additional file 1. [file 13643_2023_2177_MOESM1_ESM.docx]

Additional file 1: Characteristics of included articles in the review

| **Citation**  **(author, year)** | **Study country, income level** | **Study population** | **Sample size** | **Study design, number of intervention groups** |
| --- | --- | --- | --- | --- |
| Adebowale et al (2014) | Nigeria, Low-income | PHC workers | 80 | Pre-post (before after) with no control group quasi-experimental design, one |
| Albedaiwi et al (2005) | USA, High-income | All adult patients who visited Tuality Health Care primary care clinics between July 1, 2001 and September 30, 2001 | 3920 | The study is interventional but the specific design is not clear, one |
| Alexander et al (2013) | Kenya, low-income | Primary health care providers who provide medical services in private clinics | 44 | Randomized controlled trial, Two (intervention and wait list control) |
| Andersen et al (1990) | USA, High-income | Primary care physicians in public health system and private practice | 41 | Randomized controlled trial, Two (experimental condition and wait-list control) |
| Badger et al (1988) | USA, High-income | Family practice residents | 36 | Cluster randomized controlled trial, Two (experimental and control sites) |
| Bermejo et al (2007) | Germany, High-income | Primary care patients and primary care GPs | 43 GPs and 1045 patients | A naturalistic pre-post intervention with control group, Two groups (intervention and control) |
| Bodlund et al (1999) | Sweden, High-income | Consecutive and unselected primary care attenders and GPs | 374 patients | Follow-up pre-post study with no control group, one group |
| Callahan et al (1996) | USA, High-income | GMP physicians and their patients aged 60 years and older who visited GMP between January 1991 and July 1993 | 254 patients and 111 GPs | Randomized clinical trial, Two groups (intervention and control) |
| Christensen et al (2003) | Denmark, High-income | General practitioners (GPs) and consecutive patients presenting with a new health problem in general practices | 38 GPs and 1785 patients | Randomized controlled trial, Two groups (randomized to have the SQ disclosed or blinded to their GP) |
| Croudace et al (2003) | UK, High-income | Consecutive surgery attenders and practitioners of general practices | 116 practitioners and 1,009 patients | A pair-matched, cluster RCT  design, Two groups (guideline practices and usual care practices) |
| Davidson et al (2006) | Australia, High-income | Nursing home patients and their GPs | 13 GPs and 55 patients | Before -after with no control group design, one group |
| Davies et al (2003) | UK, High-income | Postpartum women (1, 3, 6, 12 and 18 months postpartum) in general practices | 252, 247, 202, 86 women at 1, 3, 6, 12 and 18 months postpartum | Before -after evaluation with no control group, one group |
| Diez-Canseco et al (2018) | Peru, Middle-income | Primary healthcare providers (midwives, nurses, and nurse assistants) and adult patients, aged ≥18 years and attending participating services, | 1772 patients and 22 primary health care providers | Mixed methods feasibility study, one group |
| Dwinnells et al (2015) | USA, High-income | Eligible patient visits in the intervention and control sites during the study period, | 2,482 patients self-administered the SBIRT screening tool and 1,685 comparable patients visited the control site | Quasi-experimental design (one facility as intervention site and one as control site), Two groups (patients in intervention site and patients in control site) |
| Fallucco et al (2019) | USA, High-income | Primary care providers (PCPs) and eligible adolescent well-visits that occurred at 14 different practices in the 2-year study period | 22 PCPs and 7,108 adolescent well-visits | Before-after with no control group design, one group |
| Feldman et al (2006) | USA, High-income | Primary care physicians (PCPs) | 152 PCPs | Randomized controlled trial, Two groups (Physicians were assigned randomly to see 2  SPs with depression/wrist pain or adjustment disorder/back pain) |
| Garg et al  (2019) | India, middle income | Primary care doctors (PCDs) | 23 PCDs | A pre‑ and post‑design method, one group |
| German et al (1987) | USA, High-income | Patients aged 18 years and older attending the Johns Hopkins Internal Medical Associates (JHIMA) during the study time | 1242 people agreed to participate (160 to 165 people in each group) | Randomized clinical trial, Five groups |
| Gledhill et al (2003) | UK, High-income | Adolescents aged 13–17 years consecutively attending the general practice and GPs | 200 adolescents in the first phase and over the six months following training 190 consecutive adolescents; n= 10 GPs | Before-after with no control group design, one group |
| Linn et al  (1980) | USA, High-income | General medical outpatients and physicians (the physician group consisted of house staff and faculty members of the Department of Medicine) | 150 randomized into six groups (24-26 in each group) | Randomized controlled trial, six groups (five treatment and one control) |
| Gomez-Restrepo et al (2007) | Columbia, Middle-income | General practitioners and patients | 1650 patients before and 1832 patients after | Pre-post design, one group |
| Hannaford et al (1996) | UK, High-income | General practitioners and patients, n | 3863 patients before and 3395 patients after | Pre-post design, one group |
| Jordans et al (2019) | Nepal, Low-income | PHC providers and patients | 179 before and 137 after | Pre-post design, one group |
| Kalina et al (2016) | USA, High-income | Women attending Obstetrics &Gynecology clinic after delivery | 45 before and 71 after intervention | Pre-post design, one group |
| Kozel et al (2012) | Slovenia, High-income | Family practitioners and routine care attendants | 2328 | Pre-post design, one group |
| Leslie et al (2017) | USA, High-income | Physicians and Pediatrics clinic clients | 282 before and 88 after | Pre-post design, one group |
| Lewandowski et al (2016) | USA, High-income | Routine PHC adolescents | 44,342 | Pre-post design, one group |
| Libby et al (2014) | USA, High-income | Routine PHC adolescent clients | 264 | Pre-post design, one group |
| Miller et al (2020) | USA, High-income | Antenatal care clients | 3634 before and 4805 After | Pre-post design, one group |
| Nakku et al (2019) | Uganda, Low-income | PHC providers | 1290 at baseline and 3481 at end-line for the community sample; and n=1893 at baseline, n= 2050 at midline and n= 1892 at end-line for the facility sample | Pre-post design, one group |
| Haddad et al (2018) | UK, High-income | School nurses | 81 intervention and 65 control | Quest Cluster randomized controlled trial, two groups |
| Kauye et al (2013) | Malawi, Low-income | PHC providers | 22 | Cluster randomized controlled trial, two groups |
| Kick et al (1999) | USA, High-income | Internal medicine residents | 36 | Randomized controlled trial, two groups (intervention and control) |
| Leng et al (2010) | USA, High-income | Routine PHC visiting patients | 256 | Randomized controlled trial, two groups (intervention and control) |
| Lin et al (2001) | USA, High-income | Out-patient department clients and PHC physicians | 139 clients | Randomized controlled trial, two groups (intervention and control) |
| Moore et al (1978) | USA, High-income | Adult patients attending PHC and resident physicians | Sample size not indicated | Randomized controlled trial, two groups (intervention and control) |
| Kutcher et al (2017) | Malawi, Low-income | Youth and community-based healthcare providers | 122 youth | Pre-post design, one group |
| Petersen et al 2019 | South Africa, Middle-income | Primary health care nurses and chronic care patients | 1310 pre-implementation and 1246 post-implementation | Pre-post pragmatic complex intervention, one group |
| Pond et al (1994) | Australia, High-income | All GPs conducting clinics at a large retirement village complex in Sydney and, Australia patients | 13 GPs and 200 patients | Pre-post design, one group |
| Rand et al (1988) | USA, High-income | Family practice resident physicians and consecutive eligible patients | 32 physicians and 1040 patients | Pre-post design with control group, two groups (experimental and control groups) |
| Romera et al (2013) | Spain, High income | Primary care physicians (n=69) and patients | 69 PCPs and 3414 patients | Pragmatic cluster-randomized trial, Two groups (intervention group and control group) |
| Schriger et al (2001) | USA, High-income | Physicians who work in the Emergency Department and patients who seek Emergency Department care | 218 patients | Randomized controlled trial, two groups (intervention or report group and control or no report group |
| Sherman et al (2004) | USA, High-income | Academic group practices or teams (internal medicine and psychiatry faculty, nurse practitioners, a social worker, a dietitian, clerical staff, and health care trainees) and patients | 262 pre- and 1424 post intervention | Pre- post (implementation of evidence-based  quality improvement, one group |
| Sorkin et al (2019) | USA, High-income | Primary care providers and patients | 390 patients | Cluster randomized controlled trial, two groups (intervention arm and control arm |
| Thompson et al (2000) | UK, High-income | Primary care physicians | 152 | Randomized controlled trial, Two groups (intervention and control) |
| Vandaele et al (2014) | Belgium, High-income | Home nurses | 92 | A quasi-experimental field study, two groups (intervention and control) |
| Vanos et al (1999) | Netherlands, High-income | Primary healthcare physicians and patients | 17 PCPs and 1778 patients screened using GHQ12 and 518 interviewed using CIDI-PHC at pre training; 1724 screened using GHQ12 and 498 interviewed using CIDI-PHC at post training | Pre-post design with no control group, one group |
| Whooley et al (2000) | USA, High-income | Primary care physicians and patients | 162 in the intervention group and 169 in the control group | Randomized controlled trial, Two groups (intervention and control) |
| Williams et al (1999) | USA, High-income | Health care workers (Community family physicians, faculty general internists, and internal medicine house staff) and consecutive adult patients | 969 patients | Randomized controlled trial, three groups (usual care or control group, case-finding with a single question and case-finding with the 20-item instrument ) |
| Worrall et al (1999) | Canada, High-income | Family physicians and patients with newly diagnosed depression | 42 physicians and 147 patients | Randomized controlled trial, Two groups (intervention and control) |
| Yonkers et al (2009) | USA, High-income | Pregnant and postpartum women receiving obstetrical care at publicly funded health care clinics | Group 1 367 women, Group 2 400 women and  Group 3 569 women | Pre- post design (evaluation of a program), three groups (Group 1, women assessed before the initiative began; group 2, women assessed after the initiative began and who were enrolled in the program; group 3, women assessed after the initiative began but who were not enrolled in the program) |
| Scott et al (2002) | UK, High-income | General practitioners (GPs) and patients | 5435 patients at practice A and 3250 at practice B | Before-after study, one group |
| Shirazi et al (2013) | Iran, Upper middle-income | General practitioners (GPs) and standardized patients | 384 GPs | Parallel group, randomized control trial, four groups (two intervention groups or tailored education and two control groups or conventional education) |
| Zupancic et al (2010) | USA, High-income | Consenting patients attending the clinics | pretest 244 and posttest 168 | Pre post (retrospective chart review), one group |
| Yawn et al (2012) | USA, High-income | Healthcare workers and postpartum women | 2,343 women in 28 practices | RCT (stratified and cross over), two groups (usual care and intervention group) |
| Vicente et al (2007) | Chile, High-income | Primary care physicians and patients | 37 PCPs and 1243 patients at pretest and 1346 patients at post test | Pre-post, one group |
| Upton et al (1999) | UK, High-income | General practitioners and patients | 17 general practitioners and 10036 patients before and 9152 patients after | Pre-post, one group |
| Rinke et al (2019) | USA, High-income | Health workers and patients in 43 practices | control phase 3394 patients, intervention phase 4114 patients, sustain phase, 2421 patients , and maintain phase 934 patients | Stepped-wedge cluster randomized control trial, 3 clusters each have control phase, intervention phase, sustain phase and maintenance phase |

PHC= Primary healthcare; GP= General practitioner; GMP= General medical practitioner; RCT= Randomized-controlled trial; PCPs= Primary care providers; PCDs= Primary care doctors; GHQ= General Health Questionnaire; SBIRT= Behavioral health Screening, Brief Intervention, and Referral to Treatment; SQ= Self-reporting questionnaire; CIDI= Composite International Diagnostic Interview.
