# Supplementary material for: Interventions to improve the detection of depression in primary healthcare: systematic review
Source: Syst Rev. 2023 Feb 24;12:25. doi: 10.1186/s13643-023-02177-6 (PMC9951508; doi:10.1186/s13643-023-02177-6)
Supplement: Supplementary file 2 — Additional file 2. [file 13643_2023_2177_MOESM2_ESM.docx]

Additional file 2: Assessment of the quality of included studies

| No | Author (year) | Criteria | | | | | | |
| --- | --- | --- | --- | --- | --- | --- | --- | --- |
|  |  | Selection bias | Study design | Confounders | Blinding | Data collection method | Withdrawals and dropouts | Global rating |
| 1 | Adebowale et al (2014) | Weak | Moderate | Weak | Weak | Weak | Strong | Weak |
| 2 | Albedaiwi et al (2005) | Weak | Weak | Weak | Moderate | Strong | Strong | Weak |
| 3 | Alexander et al (2013) | Moderate | Strong | Strong | Moderate | Strong | Weak | Moderate |
| 4 | Andersen et al (1990) | Moderate | Strong | Moderate | Moderate | Moderate | Weak | Weak |
| 5 | Badger et al (1988) | Moderate | Strong | Moderate | Moderate | Strong | Weak | Moderate |
| 6 | Bermejo et al (2007) | Weak | Moderate | Moderate | Moderate | Strong | Moderate | Moderate |
| 7 | Bodlund et al (1999) | Moderate | Weak | Strong | Moderate | Strong | Strong | Moderate |
| 8 | Callahan et al (1996) | Moderate | Strong | Weak | Moderate | Strong | Weak | Weak |
| 9 | Christensen et al (2003) | Strong | Strong | Strong | Strong | Strong | Strong | Strong |
| 10 | Croudace et al (2003) | Strong | Strong | Strong | Moderate | Strong | Moderate | Strong |
| 11 | Davidson et al (2006) | Weak | Moderate | Moderate | Weak | Strong | Moderate | Weak |
| 12 | Davies et al (2003) | Weak | Moderate | Weak | Weak | Strong | Weak | Weak |
| 13 | Diez-Canseco et al (2018) | Moderate | Moderate | Weak | Moderate | Weak | Weak | Weak |
| 14 | Dwinnells et al (2015) | Moderate | Moderate | Strong | Moderate | Strong | Weak | Moderate |
| 15 | Fallucco et al (2019) | Strong | Moderate | Strong | Moderate | Strong | Strong | Strong |
| 16 | Feldman et al (2006) | Moderate | Strong | Strong | Strong | Strong | Strong | Strong |
| 17 | Garg et al (2019) | Weak | Moderate | Strong | Moderate | Weak | Strong | Moderate |
| 18 | German et al (1987) | Moderate | Strong | Strong | Strong | Strong | Weak | Moderate |
| 19 | Gledhill et al (2003) | Moderate | Moderate | Moderate | Moderate | Strong | Weak | Weak |
| 20 | Linn et al (1980) | Moderate | Strong | Strong | Moderate | Strong | Strong | Strong |
| 21 | Gomez-Restrepo et al (2007) | Strong | Weak | Weak | Weak | Moderate | Moderate | Moderate |
| 22 | Hannaford et al (1996) | Moderate | Weak | Strong | Strong | Moderate | Moderate | Moderate |
| 23 | Jordans et al (2019) | Moderate | Moderate | Strong | Weak | Moderate | Moderate | Moderate |
| 24 | Kalina et al (2016) | Moderate | Moderate | Strong | Moderate | Strong | Weak | Moderate |
| 25 | Kozel et al (2012) | Moderate | Weak | Weak | Weak | Moderate | Weak | Weak |
| 26 | Leslie et al (2017) | Weak | Moderate | Weak | Weak | Strong | Moderate | Weak |
| 27 | Lewandowski et al (2016) | Moderate | Moderate | Strong | Weak | Strong | Moderate | Moderate |
| 28 | Libby et al (2014) | Moderate | Moderate | Weak | Weak | Strong | Moderate | Moderate |
| 29 | Miller et al (2020) | Weak | Moderate | Weak | Weak | Weak | Weak | Weak |
| 30 | Nakku et al (2019) | Moderate | Moderate | Strong | Weak | Moderate | Moderate | Moderate |
| 31 | Haddad et al (2018) | Moderate | Strong | Strong | Weak | Moderate | Moderate | Moderate |
| 32 | Kauye et al (2013) | Strong | Strong | Strong | Strong | Strong | Strong | Strong |
| 33 | Kick et al (1999) | Strong | Strong | Weak | Weak | Moderate | Moderate | Moderate |
| 34 | Leng et al (2010) | Moderate | Strong | Strong | Weak | Strong | Moderate | Strong |
| 35 | Lin et al (2001) | Moderate | Strong | Strong | Strong | Weak | Weak | Moderate |
| 36 | Moore et al (1978) | Moderate | Strong | Weak | Weak | Strong | Weak | Moderate |
| 37 | Kutcher et al (2017) | Weak | Weak | Strong | Weak | Strong | Moderate | Weak |
| 38 | Petersen et al 2019 | Moderate | Weak | Strong | Moderate | Moderate | Strong | Moderate |
| 39 | Pond et al (1994) | Moderate | Weak | Strong | Weak | Strong | Strong | Moderate |
| 40 | Rand et al (1988) | Strong | Weak | Strong | Moderate | Strong | Strong | Moderate |
| 41 | Romera et al (2013) | Moderate | Moderate | Strong | Weak | Strong | Moderate | Moderate |
| 42 | Schriger et al (2001) | Moderate | Strong | Strong | Strong | Strong | Strong | Strong |
| 43 | Sherman et al (2004) | Moderate | Weak | Weak | Moderate | Strong | Weak | Weak |
| 44 | Sorkin et al (2019) | Moderate | Moderate | Weak | Moderate | Strong | Strong | Moderate |
| 45 | Thompson et al (2000) | Strong | Strong | Strong | Moderate | Strong | Strong | Strong |
| 46 | Van Daele et al (2014) | Moderate | Weak | Weak | Moderate | Strong | Strong | Weak |
| 47 | Vanos et al (1999) | Weak | Weak | Strong | Moderate | Strong | Moderate | Moderate |
| 48 | Whooley et al (2000) | Strong | Strong | Weak | Strong | Strong | Strong | Strong |
| 49 | Williams et al (1999) | Strong | Strong | Strong | Strong | Strong | Strong | Strong |
| 50 | Worrall et al (1999) | Moderate | Strong | Strong | Moderate | Strong | Strong | Strong |
| 51 | Yonkers et al (2009) | Moderate | Weak | Strong | Moderate | Strong | Moderate | Moderate |
| 52 | Scott et al (2002) | Weak | Weak | Weak | Moderate | Strong | Weak | Weak |
| 53 | Shirazi et al (2013) | Strong | Moderate | Strong | Strong | Strong | Strong | Strong |
| 54 | Zupancic et al (2010) | Weak | Weak | Strong | Moderate | Strong | Weak | Weak |
| 55 | Yawn et al (2012) | Strong | Moderate | Strong | Moderate | Strong | Moderate | Moderate |
| 56 | Vicente et al (2007) | Moderate | Weak | Weak | Moderate | Strong | Weak | Moderate |
| 57 | Upton et al (1999) | Moderate | Weak | Strong | Moderate | Strong | Strong | Moderate |
| 58 | Rinke et al (2019) | Strong | Strong | Strong | Moderate | Strong | Moderate | Moderate |
