# Supplementary material for: Interventions to improve the detection of depression in primary healthcare: systematic review
Source: Syst Rev. 2023 Feb 24;12:25. doi: 10.1186/s13643-023-02177-6 (PMC9951508; doi:10.1186/s13643-023-02177-6)
Supplement: Supplementary file 3 — Additional file 3. [file 13643_2023_2177_MOESM3_ESM.docx]

Additional File 3: Search strategy (for the PubMed database)

- Depression OR “depressive disorder” OR “major depressive disorder” OR “minor depression” OR “bipolar depression” OR “masked depression” OR “secondary depression” OR “sub-threshold depression” OR “mild depression” OR dysthymia OR “common mental disorder”

AND

- Detection OR “detection rate” OR screening OR “case finding” OR diagnosis OR undiagnosed OR under-detection OR recognition OR “under recognition”

AND

- “Primary health care” OR “primary care” OR “health center” OR “primary hospital” OR “community health” OR “Family practice”

AND

- Intervene* OR strategy* OR method* OR mechanism* OR screening OR guideline OR education OR “clinician education” OR “case management” OR “collaborative care” OR “stepped care” OR “chronic care model”
